# Investigation on Effect of Strain Rate and Heat Generation on Traverse Force in FSW of Dissimilar Aerospace Grade Aluminium Alloys

**DOI:** 10.3390/ma12101641

**Published:** 2019-05-20

**Authors:** Noor Zaman Khan, Dhruv Bajaj, Arshad Noor Siddiquee, Zahid A. Khan, Mustufa Haider Abidi, Usama Umer, Hisham Alkhalefah

**Affiliations:** 1 Department of Mechanical Engineering, National Institute of Technology, Srinagar 190006, Jammu and Kashmir, India; noor_0315@yahoo.com; 2 Department of Mechanical Engineering, Jamia Millia Islamia (A Central University), New Delhi 110025, India; maildhruv08@gmail.com (D.B.); zakhan@jmi.ac.in (Z.A.K.); 3 King Saud University, Advanced Manufacturing Institute, Riyadh 11421, Saudi Arabia; uumer@ksu.edu.sa (U.U.); halkhalefah@ksu.edu.sa (H.A.)

**Keywords:** aluminium alloys, friction stir welding, strain rate, traverse force

## Abstract

The emergence of the aerospace sector requires efficient joining of aerospace grade aluminium alloys. For large-scale industrial practices, achievement of optimum friction stir welding (FSW) parameters is chiefly aimed at obtaining maximum strain rate in deforming material with least application of traverse force on the tool pin. Exact computation of strain rate is not possible due to complex and unexposed material flow kinematics. Estimation using micro-structural evolution serves as one of the very few methods applicable to analyze the yet unmapped interdependence of strain rate and traverse force. Therefore, the present work assessed strain rate in the stir zone using Zener Holloman parameter. The maximum and minimum strain rates of 6.95 and 0.31 s^−1^ were obtained for highest and least traverse force, respectively.

## 1. Introduction

Friction stir welding (FSW) of aluminium alloys has opened new avenues of building lightweight and yet high strength structures. This process overcomes various problems associated with conventional fusion welding processes in joining of high strength aluminium alloys. Benefits of FSW over conventional fusion welding processes include the absence of brittle inter-dendritic phases in the weld microstructure [1], low distortion, improved mechanical properties of the joint and good dimensional stability [2]. FSW, invented in 1991 by W.M. Thomas at TWI [3], finds numerous applications [4,5,6,7] in a short time span compared to other welding processes. However, a coherent understanding of FSW process has not yet been achieved and there persists the quest for even better understanding of various aspects of the process, such as strain, strain rate, evolution of process forces, etc.

In addition, owing to various desirable properties such as good formability, high plasticity [8] and high strength to weight ratio [9], the usage of aluminium has increased significantly in various engineering applications [10]. Considering (1) growing applications of FSW, (2) increased usage of aluminium across various industrial sectors and (3) the need for thorough and more comprehensive understanding of the process, FSW of aluminium alloys was chosen for the subject of the present study.

FSW is a solid-state welding process [11], in which a non-consumable rotating tool with a pin is plunged in the abutting surfaces of the base materials (BM). After the plunge is complete, dwell time may be provided to the tool before the tool’s traverse in order to preheat the BM [12]; and then the rotating tool is traversed along the joint line. Heat generated by friction and plastic deformation softens the BM. The softened material is stirred by the tool pin and then consolidated behind the tool, forming a solid phase joint. According to numerical simulations, about 2 to 20% of total heat is obtained from plastic deformation [13,14], and the rest of the heat is obtained from frictional heating due to rubbing of the tool with the BM.

Plastic deformation due to stirring induces severe plastic deformation (SPD) in the BM at a very high strain rate. During plastic deformation, shear stress plays an important role in determining the material flow characteristics. Shear stress induced in the BM during FSW is also a key topic which needs further understanding. However, shear stress in the BM also varies with strain rate [12]. Thus, studies based upon strain rate undergone by the material are very important for the coherent understanding of FSW process.

Due to plastic deformation of the material by traversing the tool, a force, termed as traverse force, is experienced by the tool in the direction opposite to tool movement. High process torque and traverse forces may lead to shear failure of the tool pin. Since tool pin failure is the most common cause of tool failure in FSW [15], understanding of traverse force holds significance for the development of tool materials, tool design and the application of FSW in manufacturing industries involving high strength materials. Su and Wu [16] estimated traverse force during FSW using radius of the recrystallized zone for different pin profiles using calculated strain rate. They found that tool pin profiles significantly affect the strain rate, plastic deformation and traverse force exerted on the base metal.

Experimental investigations and numerical analysis have been performed by researchers in order to estimate the temperature fields, strain and strain rate during FSW [17,18,19,20,21,22]. Frigaard et al. [23] used electron backscattered diffraction (EBSD) technique and a three-dimensional heat flow model to estimate the strain rate. Strain rates of 1–2 and 10–18 s^−1^ were reported for non-recrystallized and recrystallized region, respectively. Masaki et al. [22] simulated the recrystallized micro-structure of aluminium alloy using plain strain compression and estimated a strain rate of 1.8 s^−1^ during FSW. A combined three-dimensional heat transfer and visco-plastic flow model was used by Nandan et al. [24] and Arora et al. [25] to compute strain rate during FSW. Detailed variation of strain rate was computed at different depths along the weld thickness by Nandan et al. [24]. Kumar et al. [26] used particle image velocimetry to measure the strain rate around the tool pin during FSW. A transparent visco-plastic material consisting of micro-glass tracers was used in this study. Maximum strain rate of 20 s^−1^ was observed using this method and an attempt was made to predict strain rate variation with respect to change in tool traverse and rotational speeds. Chen and Cui [27] used a broken pin embedded into the work-piece in order to estimate the strain and strain rate ahead of the tool pin during FSW. A strain of 3.5 and strain rate of 85 s^−1^ was calculated by analyzing the deformed dendrites. Luo et al. [28] analyzed the welding characteristics of 2A14-T6 aluminum alloy using FSW. Tensile testing shows that the strength coefficient of the joint reaches 82.5%. Dong et al. [29] studied the micro-structures of the joints of dissimilar aluminium alloys (AA7003-T4 and 6060-T4) prepared using FSW. It was concluded that the weak area exists in the heat-affected zone (HAZ) of 6060 alloy, which was placed in the retreating side during FSW.

It is pertinent to mention that the material flow and joint consolidation closely relate to the flow stress, which also depends on the prevailing temperature. Further, the prevalent strain rate, in turn, also affects the flow stress. Under this situation the material flow and process forces become significantly dependent on the strain rate and welding temperature. Incidentally, high temperature is detrimental to the strength of age-hardened material, while low temperature makes the material flow difficult. Importantly, the specific relation between the traverse force and shear stress with regard to the FSW is not available and this becomes the motivation for conducting the present research work by correlating the strain rate and process force at different welding conditions. Though several researchers have experimentally measured and analyzed traverse force exerted on the tool [30,31,32], and induced strain rate [22,23,24] during FSW, the available literature on the inherent correlation between traverse force and strain rate is scarcely reported. In the present paper, the relationship between traverse force and strain rate has been studied with reference to two important FSW parameters, i.e., tool traverse speed and tool rotational speed.

## 2. Materials and Methods

Aluminium alloys AA2219-O and AA7475-T761 were selected as base materials for welding. The chemical composition of AA2219 and AA7475 alloys is given in Table 1 and Table 2, respectively. Mechanical and thermal properties of the base materials are shown in Table 3.

**Figure 1:**
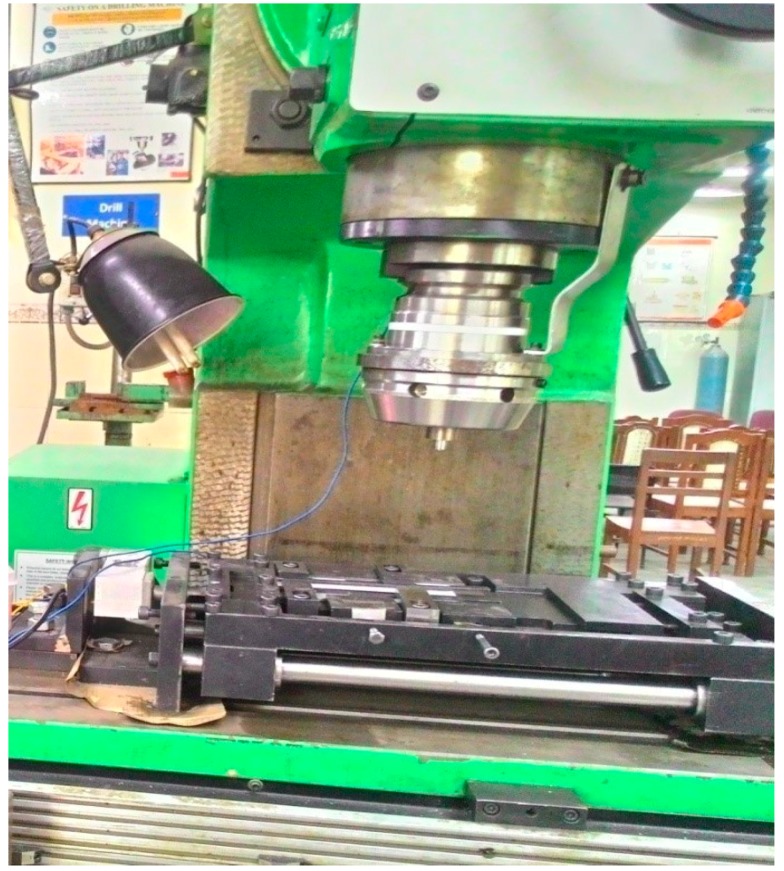
Experimental setup used for performing friction stir welding.

Welding experiments were performed on a robust vertical milling machine adapted for performing FSW. A cylindrical tool having threaded pin with 14 mm shoulder diameter and 4 mm pin diameter was used for performing FSW. High carbon high chromium steel was selected as the tool material. Tool tilt angle and tool insertion were kept constant at 2.5° and 2.25 mm, respectively. 2.5 mm thick plates of AA2219 and AA7475 alloys were welded in butt-joint configuration. AA2219 and AA7475 alloys were kept on the advancing side (AS) and the retreating side (RS), respectively. Traverse force was measured using load cells attached with a computer interfaced data acquisition system. Customized data acquisition system was devised for recording and plotting real-time values of traverse force with distance traversed by the tool. The traverse force during FSW is strongly dependent upon the contact area between the tool and the deforming material [15]. Moreover, for the same pin length, the optimum plunge depth during FSW is dependent upon the shoulder diameter of the tool [33]. Even minor variation in plunge depth significantly affects the temperature and traverse force during welding. Therefore, the variation in shoulder diameter could prove detrimental to the rationality of results in the present analysis. Hence, only the traverse speed and the rotational speed of the tool were varied. FSW experimental plan is shown in Table 4. The set of tool traverse and rotational speeds was carefully devised after thorough trial experimentation. For metals, the temperature dependence of flow stress is stronger as compared to the dependence on strain rate [34]. Thus, in order to enhance the effect of strain rate, a large difference of about 57% with respect to lower level was selected for both traverse and rotational speed. Simultaneously, a relatively smaller but continuous increase in temperature is also achieved at this set of traverse and rotational speeds. This is required to accomplish the cumulative effect of heat generation and strain rate. In conjunction, these parameters are also able to yield sound welds. Experimental setup for FSW and force measurement is shown in Figure 1.

Wire electrical discharge machine (WEDM; manufacturer—Steer Corporation, City—Shanghai, Country—China) was used for cutting specimens for micro-structural examination. After the standard metallographic procedure, Keller’s reagent was used for etching. Etched specimens were observed under an optical microscope and mean sub-grain size was estimated according to ASTM E 112 [35] using line intercept method through a MIAS software (Version 2.0) [36].

## 3. Results and Discussion

Traverse force is an indirect measure of flow-stress required for plastic deformation of the material during FSW. Flow-stress in the deforming material depends upon (1) heat input per unit weld length and (2) the strain rate experienced by the deforming material. Higher heat input softens the material and, hence, reduces flow-stress. On the other hand, higher strain rate increases flow-stress [20]. The SPD produced due to high strain rates during stirring produces ultrafine grains through dynamic recrystallization and consequently enhances the joint strength. Thus, greater strain rate yet lower traverse force on the tool is desirable for FSW. This is because higher load augments wear, deformation and degradation of the tool, causing possible weld contamination and increased tool replacement frequency [37].

### 3.1. Estimation of Local Strain Rate

Due to the complexities involved during stirring and material flow during FSW, exact calculation of actual strain rate is very difficult. However, local strain rate can be estimated using the following relationship described by McQueen and Jonas for aluminium alloys [38]:

(1) $$d_{s}={\left[-0.6+0.08\log_{10}Z_{h}\right]}^{-1}$$

where $d_{s}$ is the mean sub-grain diameter and $Z_{h}$ is the Zener Holloman parameter given by:

(2) $$Z_{h}=\dot{\varepsilon }\;\exp\left(\frac{Q}{RT_{p}}\right)$$

**Figure 2:**
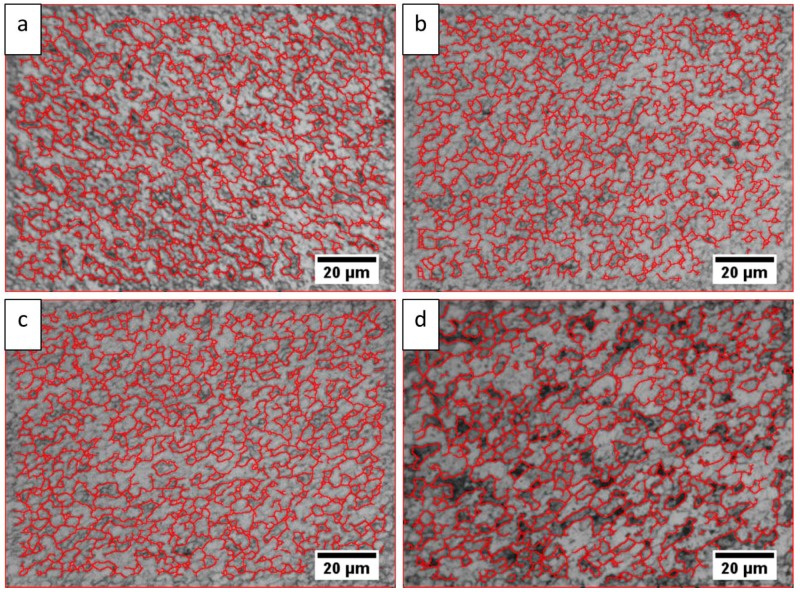
Optical Micro-graphs at region A for grain size measurement of (**a**) Experiment 1, (**b**) Experiment 2, (**c**) Experiment 3, (**d**) Experiment 4.

Here, $\dot{\varepsilon }$ is the strain rate and $T_{p}$ is the peak temperature (in Kelvin) reached during FSW at the given location, *Q* is the apparent activation energy (taken as 156 kJ/mol [23]) and *R* is the universal gas constant (8.314 J/mol). The average grain size and peak temperature of the stir zone of four welded samples was measured using the line intercept method (Figure 2) and customized thermocouple, respectively. Due to SPD, only equi-axed grains are observed in the stir zone (SZ) unlike the bi-model sized grains of the thermo-mechanically affected zone (TMAZ) and heat affected zone (HAZ). Thus, nominal grain size was used for representing the results. Morphology of grains is also dependent upon the section of the weld under analysis. However, microstructural analysis of sections parallel to the traverse force can result in noise and needs extensive care. Notably, transverse cross-section of the welded joints was analyzed in this study.

**Figure 3:**
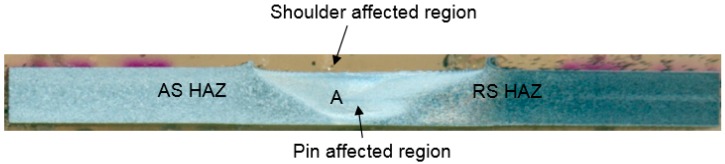
Schematic diagram showing the region A where optical micro-graphs are analyzed and local strain rate is computed.

Using the measured mean sub-grain diameter and peak temperature, local strain rate is estimated (at region A indicated in Figure 3) and presented in Table 5.

**Figure 4:**
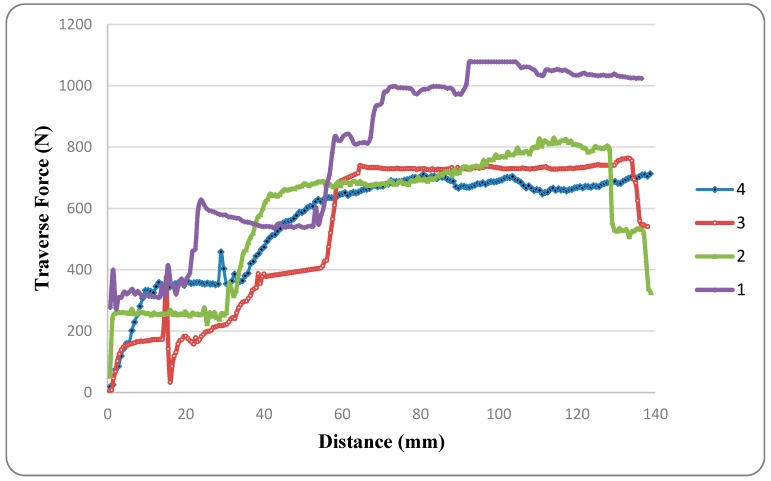
Traverse force exerted on the tool pin vs. distance travelled by the friction stir welding (FSW) tool for Experiments 1–4.

Table 6 shows the peak and average (for the specific distance of 60 mm (from 60 to 120 mm) values of traverse force exerted by the deforming material on the tool pin for different welding parameters. Figure 4 shows the variation in traverse force exerted on the tool with distance traversed for different welding parameters.

Experimental data was analyzed using standard statistical software Minitab-17 [39]. Lower the better (minimization) criterion was chosen for traverse force as its lower value results in less tool wear, low load on machine spindle and work fixture, etc. ANOVA was performed on the values of Table 7 to assess the significance of FSW parameters on traverse force and its results are given in Table 8. Results of ANOVA reveal that traverse speed is found to be the most dominating factor affecting traverse force with percentage contribution of 63.26%.

For a fixed traverse speed, higher rotational speed results in greater heat generation per unit weld length and increased material stirring action induced by the tool and thus, greater shear strain rate. However, enhanced strain rate strengthening induced in the material may not significantly contribute to the force acting along traverse direction. This is due to the fact that any change in force due to the strain rate strengthening on the tool normal to its axis is symmetrically distributed in plane normal to tool axis. Thus, change in net force in traversing direction may not significantly depend on strain rate strengthening.

Furthermore, high heat input associated with increased rotational speed induces material softening which dominates strain rate strengthening. Thus, increase in the tool rotational speed leads to decrease in flow-stress. By increasing rotational speed from 710 to 1120 rpm, the peak traverse force reduced from 1078 to 830 N (at 250 mm/min) and 764 to 705 N (at 160 mm/min), whereas average traverse force reduced from 999 to 737 N (at 250 mm/min) and 729 to 678 N (at 160 mm/min) which is evidence of reduction in flow stress.

Conversely, at fixed rotational speed, higher traverse speed results in lower heat input per unit weld length and higher strain rate. Both of these effects contribute to increase in flow-stress. Moreover, due to the increased traversing speed, the net traverse force acting on the tool witnesses a sharp increase.

Even though for Experiments 2 and 3 the change in traverse speeds is much lower than the change in rotational speeds, the traverse force in Experiment 2 was higher than that in Experiment 3. This is due to the dominant effect of traverse speed on traverse force; because apart from the heat input, drag force for the tool’s traverse adds to the traverse force. However, it can be observed from Figure 4 that, for a small displacement due to tool traverse (60–85 mm), the traverse force for Experiment 3 exceeds that of Experiment 2. This is due to the fact that excess heat was generated due to higher rotational speed in Experiment 2 during the first half of the tool traverse. This excess heat overcomes the cooling effect provided by greater traverse speed for a small distance of tool traverse.

Maximum traverse force is obtained for the combination of higher traverse speed and lower rotational speed. This is because both higher traverse speed and lower rotational speed increase the flow-stress due to lower heat input per unit length and increased traverse speed. Due to similar reasons, the peak value of traverse force is lowest for the combination of lower traverse speed and higher rotational speed.

During experimentation, before the beginning of the tool traverse, a dwell time is given during which the rotating tool remains stationary at its position after plunging. Due to the plunging and the dwell time provided, the material ahead of the tool gets pre-heated. This leads to the softening of material in the vicinity of the rotating tool. As a result, the traverse force is less in the initial phase of the tool traverse and increases as the tool progresses towards the relatively colder material as shown in Figure 4.

### 3.2. Micro-Structure

The micro-structural analysis of welded joints having dynamically re-crystallized grains provides an important means to analyze the degree of severe plastic deformation for different welding parameters. Dynamic re-crystallization in stir zone is a consequence of plastic deformation and high temperature experienced by the material during FSW. Deformation, recovery and recrystallization of the base material, which occur during FSW, lead to dynamic recrystallization. The rate of transformation of sub-grains to grains decreases and grain growth rate increases as temperature rises above recrystallization point. In addition, the increase in degree of plastic deformation/strain causes greater grain refinement in the stir zone [40].

**Figure 5:**
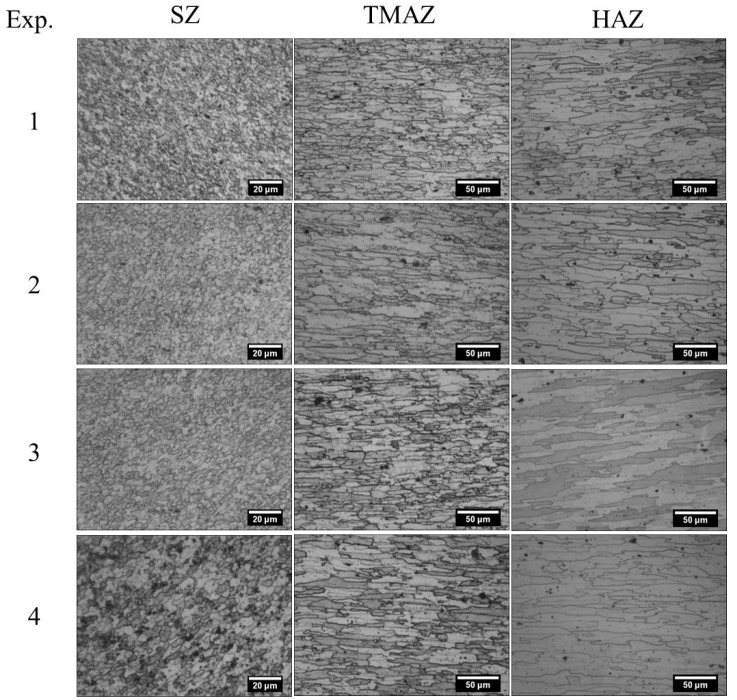
Micro-structural characterization for Experiments 1–4.

Figure 5 shows the micro-structures of the SZ, TMAZ and HAZ for all experiments. Table 9 represents the grain sizes of TMAZ and HAZ corresponding to these micro-graphs. Since grains of bi-model size exist in the TMAZ and HAZ, the grain size has been measured along the transverse/rolling direction for a comparative analysis. The strain experienced by the base metal decreases on moving from AS to RS [40]. Thus, the effect of strain rate decreases on moving from AS to RS. Thereby, estimation of strain rate was performed from the region A of the AS SZ, as shown in Figure 2.

In the SZ, maximum and minimum grain diameter of 4.8 and 2.7 µm were observed in Experiments 4 and 1, respectively. This might be due to (1) higher heat generation in Experiment 4 resulting in lower nucleation rate and higher growth rate; or (2) higher strain rate in Experiment 1 resulting in an increase in nucleation and transformation rate, resulting in smaller grain size. These results are in agreement with the work of Bird et al. [41]. Grain sizes ranging from 31.6 to 36.6 µm in the TMAZ, and 43.8 to 61.0 µm in the HAZ have been observed. Partial recrystallization in the TMAZ and induced strain from the SPD in SZ is the reason for a smaller grain size in SZ and TMAZ as compared to that of HAZ. Moreover, grain coarsening in the HAZ also contributes to the higher grain size in this zone, as shown in Table 9. The difference in the grain size of successive experiments is least for the SZ and maximum for the HAZ. This is due to the fact that prevalence of heat generation factor increasingly dominates the grain refinement due to strain as the distance from the weld center increases. However, similar order of grain size has been observed for TMAZ and HAZ for Experiments 1 to 4. The exact opposite order of magnitudes of strain rate and grain size as shown in Table 5 reflects the inherent inverse relation between the grain size and degree of plastic deformation.

## 4. Conclusions

This work has attempted to estimate strain rate through measured values of grain size and welding temperature and establish its correlation with flow stress. Optical microscopy and Zener Holloman parameter were used for the estimation of local strain rate. The following conclusions are drawn in light of the results of the conducted experimental investigations:

(1) Greater strain rate leads to the higher flow stress required for plastic deformation; however, the dependence of strain rate on tool traverse speed is more severe as compared to tool rotational speed.
(2) Effect of rotational speed on flow stress with regard to frictional heating is dominant compared to that with regard to strain rate strengthening.
(3) Maximum traverse force of 1078 N was observed at higher strain rate and vice versa.
(4) Strain rate during FSW can vary between 0.31 to 6.95 s^−1^. The same order of traverse force and estimated local strain rate (at the stir zone) for all the experiments is found. This suggests that a permissible window of strain rate can be estimated to prevent the shear failure of the tool during FSW.

Future work will include the computational simulation of the FSW process, and the results of the finite element simulation will be validated with the experimental results.

## Figures and Tables

**Table 1:** Chemical composition of AA2219-O.

Elements	Al	Cu	Sn	Mn	Fe	Si	Ti	Zn	Ni	Zr	V
**AA2219 (wt.%)**	91.97	6.8	0.02	0.315	0.16	0.06	0.04	0.06	0.023	0.203	0.165

**Table 2:** Chemical composition of AA7475-T761.

Elements	Al	Cu	Mg	Mn	Fe	Si	Ti	Zn	Ni	Zr	Cr
**AA7475 (wt.%)**	90.99	1.34	1.93	0.006	0.101	0.06	0.023	5.36	0.002	0.0076	0.155

**Table 3:** Mechanical and thermal properties of AA2219-O and AA7475-T761 alloys.

Aluminium Alloy	UTS (MPa)	Yield Strength (MPa)	Specific Heat Capacity (J/g°C)	Thermal Conductivity (W/mK)	Incipient Melting-Liquidus Temperature (°C)
**AA7475-T761**	468	430	0.88	163	538-635
**AA2219-O**	256	210	0.864	120	543–643

**Table 4:** Experimental plan.

Experiment No.	Tool Rotational Speed (rpm)	Tool Traverse Speed (mm/min)
1	710	250
2	1120	250
3	710	160
4	1120	160

**Table 5:** Estimated local strain rates and sub-grain diameter.

Experiment	$d_{s}$ (μm)	$T_{p}$ (°C)	$Z_{h}$	$\dot{\varepsilon }$(s^−1^)
1	2.5	426	3.16 × 10^12^	6.95
2	3.1	448	3.41 × 10^11^	1.69
3	3.4	462	1.50 × 10^11^	1.23
4	4.8	495	1.27 × 10^10^	0.31

**Table 6:** Peak values of traverse force for different welding parameters.

Experiment Number	Tool Rotational Speed (rpm)	Traverse Speed (mm/min)	Peak Value of Traverse Force (N)	Average Values of Traverse Force (N)
1	710	250	1078	999
2	1120	250	830	737
3	710	160	764	729
4	1120	160	705	678

**Table 7:** Experimental values for ANOVA.

Experiment Number	Tool Rotational Speed (rpm)	Traverse Speed (mm/min)	Peak Traverse Force (N)
1	710	250	1078
2	1120	250	830
3	710	160	764
4	1120	160	705

**Table 8:** Analysis of variance.

Source	Sum of Squares	DF	Mean Square	F Value	% Contribution
***A***	2.2036	1	2.2036	3.56	28.69
***B***	4.8582	1	4.8582	7.86	63.26
**Residual**	0.6184	1	0.6184		8.05
**Total**	7.6802	3			

**Table 9:** Grain size of thermo-mechanically affected zone (TMAZ) and heat affected zone (HAZ) for Experiments 1–4.

Exp. No.	Grain Size (µm)	
	TMAZ	HAZ
1	31.6	43.8
2	32.5	48.4
3	32.8	55.8
4	36.6	61.0
